# Optical coherence tomography as an alternative tool for evaluating the effects of glyphosate on hybrid catfish (*Clarias gariepinus* × *Clarias macrocephalus*)

**DOI:** 10.1016/j.toxrep.2022.01.010

**Published:** 2022-01-29

**Authors:** Chutima Thanomsit, Jadsada Saetiew, Panomsak Meemon

**Affiliations:** a Department of Fisheries, Faculty of Agriculture and Technology, Rajamangala University of Technology Isan Surin Campus, Surin, 32000, Thailand; b School of Physics, Institute of Science, Suranaree University of Technology, Nakhon Ratchasima, 30000, Thailand; c Center of Excellent in Advanced Functional Material, Suranaree University of Technology, Nakhon Ratchasima, 30000, Thailand

**Keywords:** Hybrid catfish, Glyphosate, Optical coherence tomography, Histology, Immunohistochemistry

## Abstract

Glyphosate contamination in fresh water is a major problem in agricultural countries. It affects many vital organs in freshwater organisms that are important in the food chain. Hence, the effects of glyphosate on living organism organs are of particular interest. However, several existing techniques for evaluating the effect of glyphosate on aquatic organisms require stained tissue. To study organ tissue with minimal processing, alternative technique is demanded. Here, we investigated the used of optical coherence tomography (OCT) as an alternative tool for *ex vivo* evaluation of the effect of glyphosate on organ tissues of aquatic organisms, i.e., hybrid catfish (*Clarias gariepinus* × *Clarias macrocephalus*). The targeted samples were organ tissues from the brain, gill, and liver of hybrid catfish after glyphosate exposion at concentration of 10 mg L^−1^ for 24 h. The alteration was then verified by histology, and immunohistochemistry. The study found that all three techniques provide correlated results. We observed that OCT clearly showed damage to the brain and gill tissues of glyphosate-exposed hybrid catfish. However, the alteration in liver tissue was observable but not clear for this low concentration of exposure. The results from histology and immunohistochemistry confirmed the effect of glyphosate on brain, gill, and liver tissues of hybrid catfish. The results suggest that all three techniques could be used to examine the effects of glyphosate exposure in hybrid catfish. However, the choice of a suitable technique depends upon the purpose of the study.

## Highlights

• The use of OCT to evaluate the effects of glyphosate on hybrid catfish was studied.
• OCT can capture tissue damages in hybrid catfish caused by glyphosate.
• OCT entails a short preparation time, simple analysis, and chemical free.
• OCT has potential for real-time or field-based study in aquaculture.

## Graphical abstract

**Figure:**
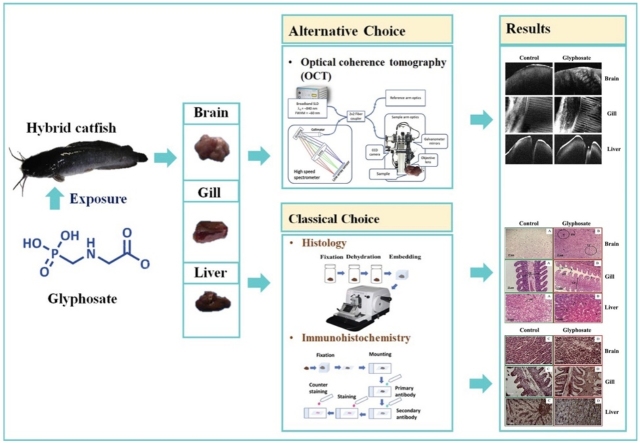

## 1 Introduction

Glyphosate was first synthesized in 1950 by Swiss scientist Dr. Henri Martin. At the time, its effectiveness in controlling weeds was not well understood. It was a scientist at Monsanto Company who later developed and used glyphosate as an herbicide. Glyphosate (C_3_H_8_NO_5_P) is an organic substance applied globally as herbicide. Roundup is a well-known trading name. Its structure consists of phosphorus in phosphonomethyl glycine group. Although glyphosate is banned in many countries, it occupies top rank among imported agro-chemicals in Thailand. Inevitably, its application causes contamination in both surface and groundwater. Many studies revealed that glyphosate contamination in water causes adverse effects on aquatic organisms. This can be biologically accumulated and magnified through food chain to human [1]. For example, in *Anguilla anguilla*, DNA was found to be damaged after exposure to glyphosate [2]. Furthermore, erythrocytes and gill cells in *Prochilodus lineatus* exposed to glyphosate were significantly higher than those in control populations [3]. In some cases, acetylcholinesterase (AChE) activity in the brain and muscle was inhibited, and the number and type of blood cells were altered [4,5]. Evaluating the effects of glyphosate can be achieved using various methods depending on the available samples, sample types, tools, and expertise. Many reports that have studied exposure to toxic substances in glyphosate herbicides are based on biomarkers and effects.

Histological changes have been widely used as biomarkers in both laboratory and field studies in the evaluation of the health of biological organisms exposed to contaminants. For field studies, histological alteration is a time-efficient method for investigating both acute and chronic exposure in tissues [[6], [7], [8]]. One of the great advantages of using histological biomarkers in environmental monitoring is that it allows for examining specific targeted organs, e.g., gills, kidney, and liver, that are responsible for vital functions, such as respiration, excretion, accumulation, and biotransformation of toxicants. Furthermore, alterations found in these organs are normally easier to identify than functional alterations and serve as early warning signs of damage to animal health [9]. Histological alterations can also detect the effects of irritants on exposed tissue and organs.Therefore, many characteristics of tissue, cell, and microscopic structures are studied using histologic methods, e.g., forensic investigation, autopsy, and diagnosis. Furthermore, histology is used widely in medicine, especially to aid in treatment of diseased tissue [10].

Structural alterations in exposed fish have also been studied by many researchers [11]. In 2013, Reddy and Rawat indicated that histological methods are an effective practice in defining toxicological effects [12]. However, they are expensive and time consuming. Moreover, histological testing is normally impractical in human subjects. Thus, biomarker characterization combined with a known histological distribution may fulfill the knowledge gap for localizing toxic injury to exposed organs or tissue.

In recent years, immunohistochemistry has developed into an integral technique in histological, immunological, and biochemical disciplines. Immunohistochemistry can be used in a wide range of cell or tissue antigen searches, ranging from amino acids and proteins to infectious agents and specific cellular populations. Immunohistochemistry is also commonly used to visualize the distribution and localization of specific cellular markers or components within a cell or tissue [13]. Immunohistochemistry is also an important tool for the elucidation of differential diagnoses that cannot be determined by conventional analysis using hematoxylin and eosin (H&E). Over the past decades, there have been many reports on immunohistochemistry and immunocytochemistry techniques. These reports cover its history, importance, applications, limitations, difficulties, limitations, and other aspects of research results [14] reported that histological, immunohistochemical, and ultrastructural analyses could be used to evaluate the function of the esophagogastric segment in freshwater tubenose goby *Proterorhinus semilunaris* [15]. However, similar to histology, immunohistochemistry requires complex sample preparation and expensive chemicals. In addition, sample analysis is time-intensive. Therefore, new techniques that are less invasive and require shorter time for sample preparation and analysis are in high demand.

Optical coherence tomography (OCT) is an emerging optical technology that is capable of depth cross-sectional imaging of biological tissue at micrometer-scale resolution without staining [16]. Recently, OCT has been applied to the study of morphology and anatomy of aquatic organisms [[17], [18], [19]]. An OCT image is analogous to an ultrasound image. However, OCT uses infrared light waves instead of ultrasound waves and therefore can achieve much higher resolution than ultrasound. Typically, OCT uses near-infrared light wavelengths at approximately 800–1300 nm to avoid water absorption and nondestructively penetrates deep below the tissue surface. By using the principle of low-coherence interferometry, OCT accurately measures the depth and intensity of back-reflected light from deep tissue [20].

To date, OCT can acquire cross-sectional images at a speed of more than 100 frames per second, allowing for noninvasive rapid imaging of biological samples. OCT has already been used in many fields, such as ophthalmology, tissue engineering, developmental biology, dentistry, dermatology, and urology [16]. OCT can be used to perform two-dimensional (2D) and three-dimensional (3D) depth cross-section imaging of biological tissues with micrometer-scale resolution. There are many existing applications of OCT to the study of aquatic organisms, e.g., tadpoles [21] and zebrafish [22]. Furthermore, the use of OCT as an alternative method for *in vivo* study of the internal physiology *in vivo* Antarctic krill under a wide range of environmental conditions was reported [23]. The authors reported that OCT enabled detailed studies of the internal physiology, e.g., heart and gastric areas, of *in vivo* Antarctic krill. In addition, retina studies in adult zebrafish comparing histological techniques with OCT techniques were also investigated [18]. Moreover, 3D characterization of adult zebrafish using OCT was performed to understand the morphology of the brain, an important organ in fish, without destroying the cells [22,24].

Fish health is generally considered a key indicator for the quality of aquatic ecosystems for many reasons. First, fish are ubiquitous in the vast majority of aquatic environments. Second, fish have high ecological relevance in aquatic environments due to their influences on the food web structure, nutrient cycling, and energy transfer. Fish are also an important protein source for humans. Third, the taxonomy, basic life history, and physiology of fish are generally well understood, allowing for targeted studies on internal levels of tissue contamination and early adverse effects. However, considerable variation exists among fish species, including their contaminant exposure patterns, their basic physiological features, and their response to environmental contaminants. Fish species used for aquatic health assessment need to be selected based on the potential pathways of exposure to the contaminant of concern [25].

In this work, we investigated the use of OCT as an alternative tool for *ex vivo* evaluation of the effect of glyphosate on freshwater fish. The hybrid catfish that is the offspring of *Clarias macrocephalus* crossed with *Clarias gariepinus* was chosen for the study since it is the most popular freshwater fish commercially cultured in Southeast Asia. The targeted samples were organ tissues from the brain, gill, and liver of hybrid catfish after been exposed to glyphosate at concentration of 10 mg L^−1^ for 24 h. To verify the results observed by OCT imaging data, we performed two addition techniques, i.e., histology and immunohistochemistry, on the same sample of fish’s tissues after OCT imaging. Histology and immunohistochemistry are considered as the gold standard for the study. The alteration of tissues was observed by comparing the results with those of a control group consisting of hybrid catfish not exposed with glyphosate.

## 2 Materials and methods

### 2.1 Chemicals

Glyphosate PESTANAL, analytical (CAS Number 1071−83-6, MFCD00055350) and reagents for histology and immunohistochemistry, i.e., hematoxylin and eosin (H&E) staining, were purchased from Sigma-Aldrich Co., Ltd. (Thailand). Primary antibody and secondary antibody, consisting of polyclonal antibody specific to AChE from electric eel (PAb-AChE, catalog number # 0200−0042) and goat anti-rabbit horseradish peroxidase conjugate (GAR-HRP, catalog number # ab 6741), were obtained from Bio-Rad (Thailand) and Abcam Company, Co. LTD. (Thailand), respectively.

### 2.2 Animal acclimation and glyphosate exposure

Immature hybrid catfish, aged less than 2 weeks, were purchased from private farms in Surin Province, Thailand. They were further cultured at the Department of Fisheries, Faculty of Agriculture and Technology, Rajamangala University of Technology, Isan Surin campus, Thailand, for 2 months. When the adult fish reached an average weight of approximately 120 ± 25.2 g, they were transferred to a 500-L cement tank and fed twice per day.

Briefly, 40 hybrid catfish were assigned into 4 tanks, 10 each. The catfish in one tank served as a control group. Following the triplicate treatment technique, the catfish in the other three tanks were exposed to the same concentration of glyphosate for 24 h. The water conditions were maintained at a pH of 6.7 ± 0.6, temperature of 25 ± 1.2 °C, and dissolved oxygen level of 6.8 ± 0.3 mg L^−1^. To capture the effect of glyphosate alone, we used the granular form of glyphosate. The glyphosate was prepared at a concentration of 10 mg L^−1^, which is the sublethal concentration as modified from Carmo Langiano and Martinez [26] who also used ganular form of glyphosate in their study. It should be noted that the fish used in Carmo Langiano and Martnez’s study was *P. lineatus*, which is different from the hybrid catfish in terms of skin. *P. lineatus is* in the class of scaled fish but the hybrid catfish is in the class of leather fish.

Three hybrid catfish from each tank were randomly selected and sacrificed. After immediately taken from the tank, these samples were anesthetized with benzocaine (0.1 g L ^− 1^) and then sectioned for collecting brain, liver, and gill samples. The OCT imaging was performed right after the organs extraction. After OCT imaging, the organs were then prepared for histology and immunohistochemistry. The remained catfish were terminated using a high pressure incinerator.

The raising and assessment behavior protocols were referenced from the guidelines for testing chemicals (OECD) 2014. All procedures involving animals were approved by the Committee for Biological Experimentation on Animals at Rajamangala University of Technology Isan, Thailand (ID-project 1/2561), under animal use license number Ul-03,405-2559.

### 2.3 OCT imaging

**Fig. 1:**
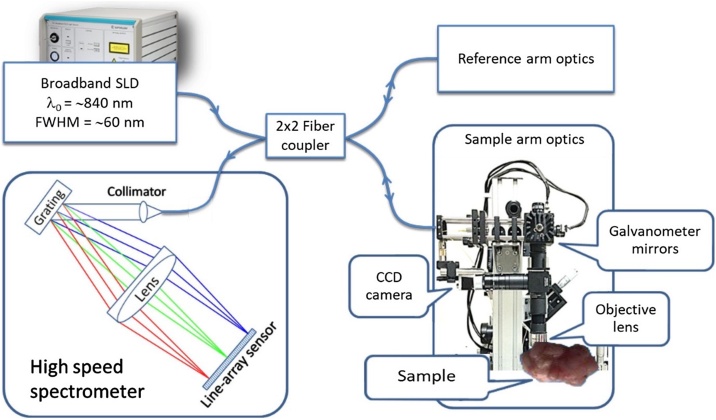
System layout of the FD-OCT imaging system.

The OCT system used in this study was a custom-built spectrometer-based frequency domain OCT (FD−OCT) system [27]. The system was designed to operate at a wavelength of approximately 850 nm. The light source was a superluminescent diode (SLD) that emitted a broad spectrum near-infrared light from 800 nm to 900 nm. The interferometer was a fiber-based Michelson interferometer, as shown in Fig. 1. The detector was a custom-built spectrometer that utilized a high-speed CMOS line sensor camera with a data acquisition speed of over 70,000 lines/second. The custom-built spectrometer allowed for high-speed image acquisition up to 100 frames per second for 1000 lines per frame of cross-sectional image. This maximum imaging speed enabled 3D data acquisition, with each dataset consisting of 1000 OCT images captured within approximately 10 s. In tissue, each cross-sectional image had an axial resolution of approximately 10 μm and transverse resolution of approximately 15 μm. The maximum penetration depth of the system was measured to be approximately 2 mm.

Since OCT imaging can be performed directly in bulk tissue without sectioning and straining, we first performed OCT imaging, followed by histology and immunohistochemistry. For collecting tissue from hybrid catfish, the process started with placing the hybrid catfish into an ice box for 10−20 min. Ice-shock was employed instead of anesthetization to ensure that the brain tissue of hybrid catfishes were not affected by chemicals. Sequentially, brain, gill and liver tissue were collected and placed in petri dishes without water to perform OCT imaging.

In this study, each 3D OCT dataset was chosen to cover a region of interest (ROI) of up to 10 mm × 10 mm laterally and approximately 1−2 mm in depth. Each 3D dataset was postprocessed in Labview (National Instrument, USA) and exported as a series of JPEG images. Using OCT images from each 3D dataset, *en face* images at different depth locations were reconstructed using ImageJ software (NIH, USA). 3D volumetric rendering was also performed using ImageJ software.

### 2.4 Histological alterations

Histological staining comprises five main steps, i.e., fixation, processing, embedding, sectioning, and staining. The most widely used staining compounds are carmine, silver nitrate, Giemsa, Trichrome stains, Gram stain, and hematoxylin [10]. In our study, after OCT imaging was performed, each tissue sample was placed in formalin fixation for 24 h. Next, samples were dehydrated through a graded series of ethanol concentrations of 50 %, 70 %, 80 %, 90 %, and absolute ethanol consecutively. Then, samples were embedded in a block of paraffin wax, and the block was prepared and sectioned at a thickness of 6 μm using a microtome (Sliding Microtomes 4004 M, A. S. Science Co, Ltd). Next, the sections were deparaffinized in xylene and stained with hematoxylin and eosin (H&E). The alterations induced by glyphosate in the tissue samples were then analyzed and photographed under a photomicroscope (CX31, Olympus Co., Ltd).

### 2.5 Immunohistochemical evaluation of AChE localization

For immunohistochemistry, a second set of slide tissue sections (6 μm) was deparaffinated and rehydrated. Then, the slides were incubated with 1% hydrogen peroxide, rinsed with distilled water, and soaked in 0.02 M phosphate-buffered saline solution with a pH of 7.4 (PBS) for 5 min. The processes were repeated 3 times. After this, the tissue slides were placed in P1^+^ solution (10 % calf serum in PBS) and incubated at 37 °C for 30 min. This was followed by applying primary antibodies specific to acetylcholinesterase (PAb-AChE) in a dilution ratio of 1:200 for 3 h at room temperature. Then, the slides were washed 4 times with PBS, for 10 min at a time to remove excess antibodies that did not react. A labeled secondary antibody (i.e., goat anti-rabbit IgG, conjugated with peroxidase; GAR-HRP dilution 1:1000) was then applied to the samples. The horseradish peroxidase label was visualized using 3,3-diaminobenzidine (DAB). The slides were soaked in the substrate solution (0.03 % DAB, 0.006 % H_2_O_2_ in PBS) for 10 min. Then, the tissues were cleared with distilled water to remove excess reactants. Finally, sections were counterstained with hematoxylin and eosin (H&E), dehydrated, and mounted with Permount.

## 3 Results and discussion

**Fig. 2:**
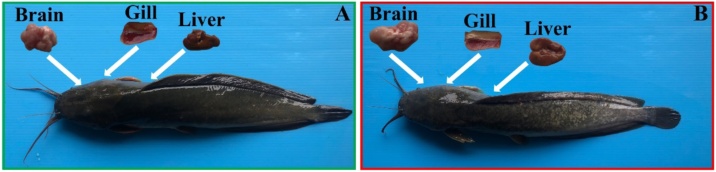
Appearance and organs (brain, gill, and liver) from hybrid catfish in the control group (A) and the glyphosate-exposed group (B).

First, we verified the effects of glyphosate on the catfish organs via visual inspection. Surgery was performed on both the control and experiment groups to dissect and separate the three major organs, i.e., brain, gill, and liver, as shown in Fig. 2. It was found that there were no visually observable differences between the organs of the hybrid catfish in the control group and the glyphosate-exposed group. This might be due to the low concentration of glyphosate or to the short exposure time. For further analysis, the harvested tissues from both the control and glyphosate-exposed groups were subjected to the three different imaging techniques as previously described.

### 3.1 Brain

Nervous and sensory systems have been a longstanding question in ecomorphology. The relationships between brain morphology and ecology are well established for select teleost fish [28]. Nevertheless, studies on the effects of glyphosate on the hybrid catfish brain are still rare. This may be due to the fact that the lipid content in the fish brain is remarkably high. The lipid content in the brain is the second highest after adipose tissue and contains essential fatty acids [29].

**Fig. 3:**
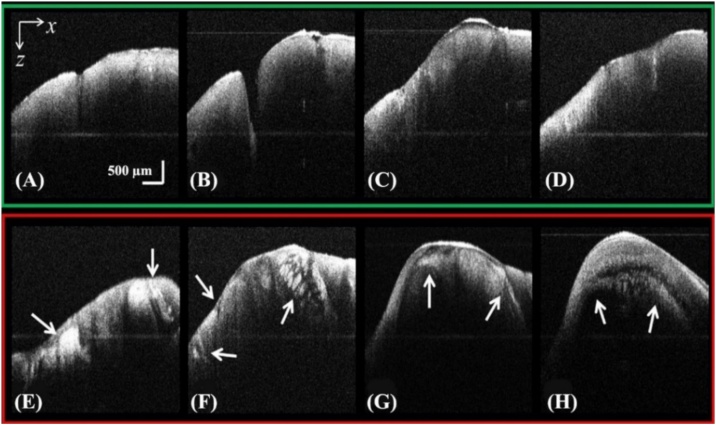
Examples of depth cross-sectional images that were captured by OCT. (A-D) and (E-H) are *XZ* cross-section images of the brain of the control hybrid catfish and the glyphosate-treated hybrid catfish, respectively.

In this study, OCT imaging was performed to evaluate both the control and the glyphosate-exposed hybrid catfish. OCT enabled observation of subsurface brain tissue without performing physical slicing, staining, and coloring, as required by histology and immunohistochemistry. The 3D dataset obtained by OCT is a series of cross-sectional images (*XZ*-plane), as shown in Fig. 3, where (A–D) and (E–H) are depth cross-section images of the control and glyphosate-treated hybrid catfish, respectively. Since the contents in brain tissue are mainly lipids, the images were expected to exhibit uniform contrast, as evidenced by the cross-sectional images of brain tissue of the control hybrid catfish in Fig. 3 (A–D). In contrast, tissue damage below the surface of the brain tissue of the glyphosate-exposed hybrid catfish is clearly observed in Fig. 3 (E–H). As indicated by the white arrows, there is severe tissue damage in the brain.

**Fig. 4:**
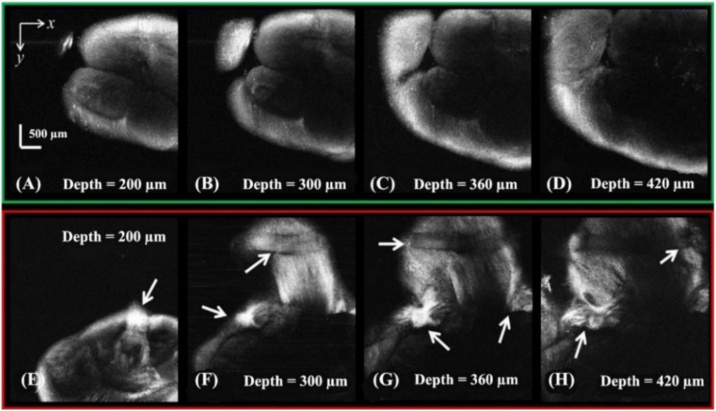
Examples of 2D *en face* images that were extracted from a recorded 3D OCT data set. (A-D) *En face* reconstructed images of the brain tissue from control hybrid catfish. (E-H) *En face* reconstructed images of the brain tissue from glyphosate-treated hybrid catfish.

Furthermore, for each captured 3D dataset, *en face* images (*XY*-plane), spaced by approximately 100 μm in depth, were reconstructed for both the control hybrid catfish and the glyphosate-treated hybrid catfish, as shown in Fig. 4 (A–D) and (E–H), respectively. This *en face* reconstruction allows for visualization of the results in the same manner as that of conventional microscopy. Again, tissue damage in the brain of the glyphosate-exposed hybrid catfish is clearly observed in Fig. 4 (E–H) when compared with normal brain tissue, as seen in Fig. 4 (A–D).

**Fig. 5:**
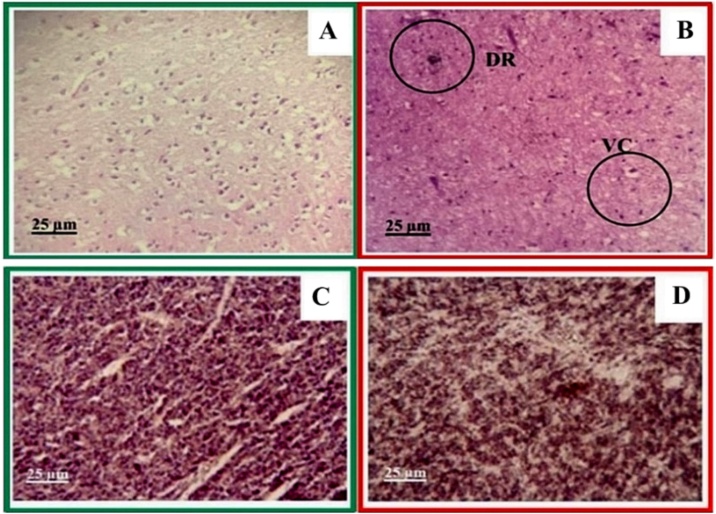
Histological alteration images of the brain tissues of (A) the control hybrid catfish compared with that of (B) the hybrid catfish exposed to glyphosate at a concentration of 10 mg L^−1^ for 24 h, where DR: degenerating neurons, VC: vacuolar changes with empty spaces that appeared as eaten away areas. (C) and (D) Results from immunohistochemistry of the control and glyphosate-exposed hybrid catfishes, respectively.

Under a light microscope to observe histological alterations of the hybrid catfish exposed for 24 h, degeneration of neurons and vacuolar changes with empty spaces appeared as eaten away areas (Fig. 5A-B). This finding was in agreement with a study on African catfish that were exposed to glyphosate for 7–28 days [28]. However, the observed effect in this study was lower than that reported in the literature since the fish were exposed to glyphosate for only 24 h and at a lower concentration of 10 mg L^−1^. For degenerating neurons, vacuolar changes with empty spaces appeared as eaten away areas. This phenomenon may indicate loss of material, which is in agreement with the study reported by Loganathan et al. [30]. However, our findings are different from those of the study performed by Ayoola, which reported that African catfish (*Clarias gariepinis*) exposed to glyphosate showed congestion, necrosis, cellular filtration spongiosis pyknosis, and hemorrhage of the neuron [31]. This difference may be due to deviations in fish species and size.

In the immunohistochemistry using a commercial polyclonal antibody at a dilution ratio of 1:100, we found brown-colored cells throughout the brain tissue, both in the control group and in the group exposed to glyphosate. However, the tissue in the fish exposed to glyphosate was darker in color. When assessing the specificity and localization of acetylcholinesterase (AChE), we found that the antibody had low specificity. This may be because the antibody used in the study was an antibody produced in electric eel, thus causing nonspecific binding (Fig. 5C-D). Moreover, the brain tissue cells were characterized by a high fat content, making them more difficult to study than other tissues. Therefore, the sample storage process before analysis was also important.

### 3.2 Gills

Despite the intense work done on the morphologic examination of gills, the organ is relatively underused in health evaluations of fish [32]. Gills play an important role in various vital functions, such as respiration, osmoregulation, and excretion. In addition, they may make contact or be exposed to the external environment or pollutants, making them sensitive to chemical and physical alterations even at low concentrations [33]. Strzyzewska et al. suggested important diagnostic guidelines for the examination of gill structure and described the morphological lesions that develop under the influence of different biological and physicochemical factors [32]. Reports show that gills are extremely sensitive to all types of handling and unfavorable changes in the external and internal environments. Therefore, studying the morphology of fish gills could provide an opportunity to assess fish health as well as gain information on possible health hazards in their environment. It has been reported that histological alteration studies can be applied to assess pollutant contamination, in particular, aquatic contamination by substances in the herbicide group.

**Fig. 6:**
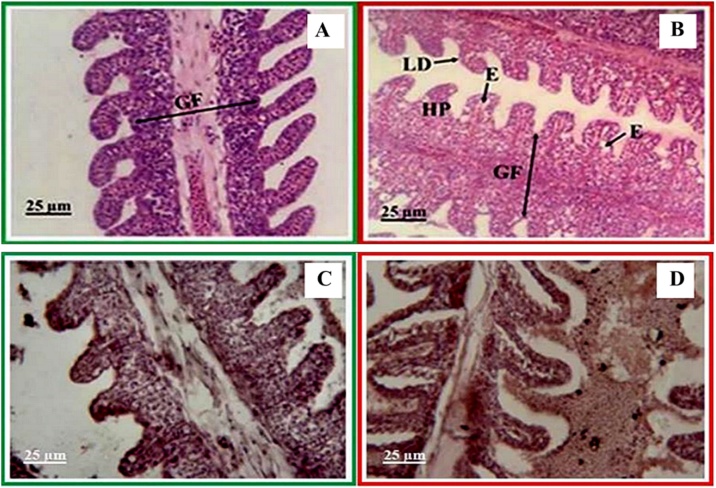
Histological alterations of gills from hybrid catfish from (A) the control group and (B) the group exposed to glyphosate at a concentration of 10 mg L^−1^ for 24 h, where GF: Gill filament, LD: Secondary lamella degeneration, E; Edema, HP: Hyperplasia. (C) and (D) are immunohistochemistry images of gills of the control and glyphosate-exposed hybrid catfishes, respectively.

This study found alterations in the form of hyperplasia, secondary lamella degeneration, and edema (Fig. 6B). Our results agree with observations in Amazon teleost fish (*Colossoma macroponum*) that showed filament hyperplasia, lamellar fusion, and edema [5] after exposure to glyphosate-based herbicides. However, that study also found filament epithelial lifting, fibrosis, and necrosis, which were not observed in our study. This difference may have been caused by the differences in exposure time. Amazon teleost fish were exposed to glyphosate for 96 h, a longer time than in our study (i.e., 24 h), and these fish showed more severe alterations. This phenomenon indicates the influence of exposure time on alterations.

Our results are consistent with our previous publication, which found that alterations in Asian sea bass were dependent on the time and concentration of glyphosate exposure [7]. These alterations can be classified into 3 types: (1) edema, fusion of lamellar irregular thickening of the primary lamellar epithelium and epithelial lifting; (2) blood congestion; and (3) lamellar aneurysm and necrosis of lamellae. In this study, we found edema and fusion of lamellae in the gill tissue, as shown in Fig. 6B. Our findings differ from those in the study of juvenile African catfish (*Clarias gariepinus*), which reported cellular infiltration, congestion, swollen tips of the gill filament, severe gill damage, and infiltration of heterophilic antibodies [31].

Furthermore, we studied immunohistochemistry by using 2 types of antibodies. The primary antibody was specific to acetylcholinesterase (AChE) from electric eel in commercial form (PAb-AChE, catalog number # 0200−0042). The secondary antibody was goat anti-rabbit horseradish peroxidase conjugate (GAR-HRP, catalog number # ab 6741). As shown in Fig. 6C and D, we found changes consistent with histology. Nevertheless, we observed brown spots on different parts of the gill tissue, which clearly showed the localization of acetylcholinesterase (AChE) expression. Namely, the areas that exhibited brown coloration were the gill lamellae and gill epithelium, which are biomarkers of exposure to glyphosate. Our observations were consistent with the observations reported by Thanomsit et al. [34].

**Fig. 7:**
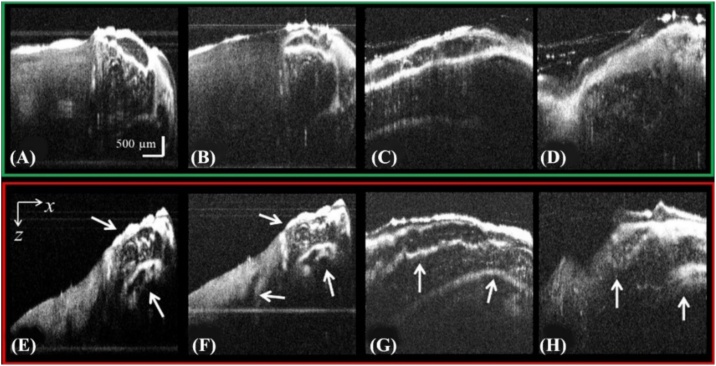
Comparison of OCT depth cross-sectional images of gill tissue obtained from (A) the control hybrid catfish and (B) the glyphosate-exposed hybrid catfish.

**Fig. 8:**
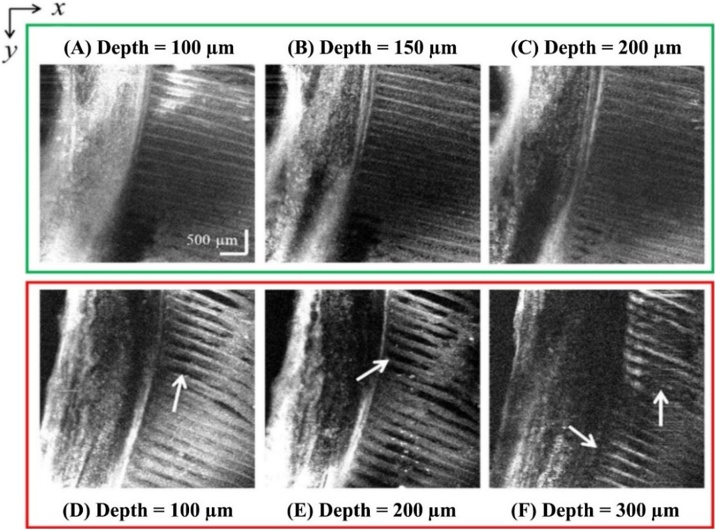
Examples of 2D *en face* images reconstructed from a 3D OCT dataset at 3 different depth locations from the surface of the gill tissue of the control hybrid catfish (A-C) compared with that of the glyphosate-treated hybrid catfish (D-F).

Fig. 7 shows OCT images of gill tissue from hybrid catfish from the control group and the group exposed to glyphosate at a concentration of 10 mg L^−1^ for 24 h. *En face* images at different depth locations from the top surface of the gill tissue of the control and experimental groups were digitally reconstructed and compared, as shown in Fig. 8. For the gill, the tissue damages are difficult to observed and interpreted in OCT cross-section images. Nevertheless, the damages can be clearly observed from the *en face* images as compared with that in the controlled group as shown in Fig. 8 (A–C). The alteration was observed as tissue loss between the bones as shown in Fig. 8 (D–F). Therefore, the OCT *en face* reconstruction is more suitable for assessment of alteration in gill tissue. It should be pointed out that, in Fig. 8, the depth locations where *en face* image were reconstructed were slightly different between the control and glyphosate-exposed group. This is to accommodate slightly different in the thickness and orientation of the acquired 3D OCT data, which should not affect interpretation of the results.

### 3.3 Liver

The organ most associated with the detoxification and biotransformation process is the liver. Because of its function, position, and blood supply, it is also one of the organs that is most affected by contaminants in the water [9]. Therefore, the liver is a good candidate for histopathological study. In 2011, Rašković et al. suggested that an important factor influencing alteration was the age of fish [35]. The indicators that are most relevant in the metabolic activity of hepatocytes and morphometric parameters are hepatocyte number, hepatocyte surface area, hepatocyte nuclear area, and the glycogen and lipid contents in the cytoplasm. After exposure to herbicide and insecticides, alterations including blood congestion, vacuolation, and necrosis of hepatocytes have been reported [7,26]. However, there are other changes such as infiltration of leukocytes, hemorrhage, and pyknosis of hepatocytes, with the severity being dependent on exposure time and concentration [31].

**Fig. 9:**
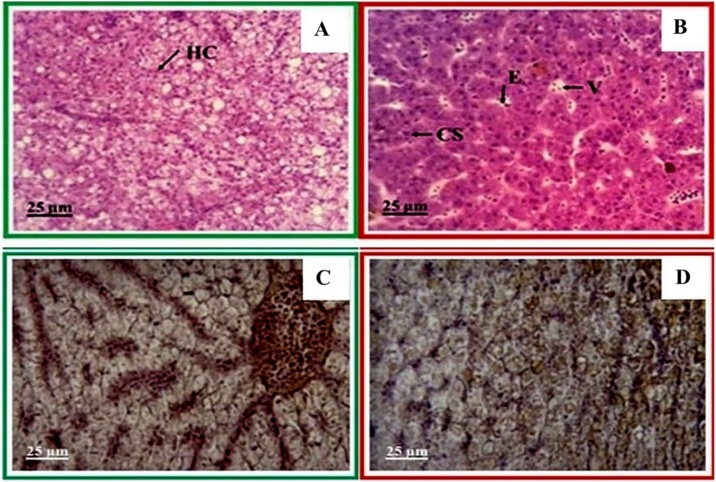
Histological alterations in the liver of (A) a hybrid catfish from the control group and (B) a hybrid catfish exposed to glyphosate at a concentration of 10 mg L^−1^ for 24 h, where HC refers to Hepatocyte, CS refers to Convergence of sinusoid, E refers to Edema, and V refers to Vacuolation (H&E, x40). (C) and (D) are immunohistochemistry images of the livers of the hybrid catfish obtained from the control group and the glyphosate-exposed group, respectively.

In this study, we report the major alterations in liver tissue found in hybrid catfish exposed to glyphosate for 24 h. Liver tissue taken from hybrid catfish in the control group, as shown in Fig. 9A, showed normal hepatocytes. This contrasted strongly with images from the 24 -h exposed group, as shown in Fig. 9B. However, it was not different from the images from hybrid catfish exposed to glyphosate for 1, 6, and 12 h (data not shown). The alterations observed included vacuolation, edema and convergence of the sinusoid, as evidenced in Fig. 9B. In addition, the immunohistochemical results of liver tissue showed a positive effect in the cytoplasmic areas for both the control group and the exposed group. The expression (dark brown color) was found in the cytoplasm of hepatocytes in liver, as shown in Fig. 9 C and D. Our results are consistent to those found by Abdulkareem et al. [36] and Thanomsit et al. [34,37].

**Fig. 10:**
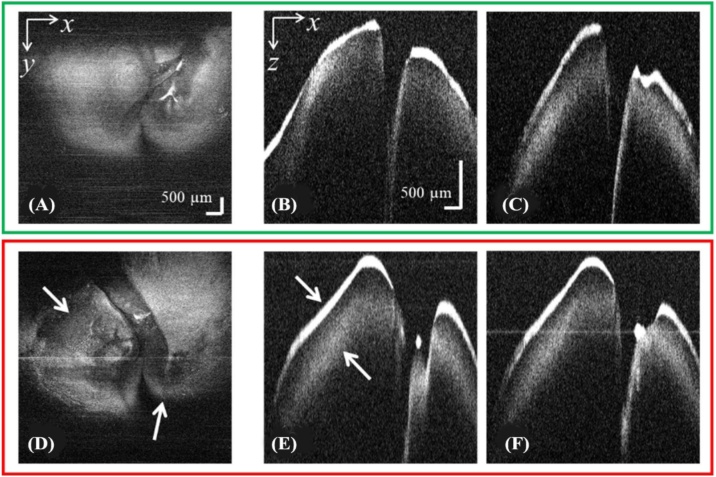
(A) *En face* images reconstructed from the 3D OCT dataset acquired from *ex vivo* liver tissue of the control hybrid catfish. (B-C) Example of depth cross-sectional images from the same 3D OCT dataset in (A). (D) *En face* images reconstructed from the 3D OCT dataset acquired from *ex vivo* liver tissue of the glyphosate-exposed hybrid catfish. (E-F) Example of depth cross-sectional images from the same 3D OCT dataset as in (C).

Fig. 10 (A) and (D) show *en face* images reconstructed from 3D OCT datasets acquired from *ex vivo* liver tissue of the control hybrid catfish and glyphosate-exposed hybrid catfish groups, respectively. Comparative tissue damage was observed in the glyphosate-exposed group. In addition, Fig. 10 (B–C) and (E–F) show OCT depth cross-sectional images of the liver tissue of the control and glyphosate-exposed fish, respectively. In the case of liver tissue, damage is not clearly observed from the cross-sectional views of the OCT images. However, we observed strong reflection at the surface of the glyphosate-exposed tissue compared with that of the control sample. Furthermore, tissue from glyphosate-exposed fish exhibited strong scattering beneath the sample surface when compared with control fish. OCT measures the amount of light scattered back from biological tissue, which is mainly governed by variation or non-uniformity of refractive index of the tissue. The harden tissue tends to exhibit higher refractive index as compared with soft tissue, which could lead to strong reflection and scattering of photon around that region [38]. Therefore, we suspect that the observed stronger light reflection and scattering beneath the tissue’s surface could be related to the hardening of the liver tissue after exposure to glyphosate. As part of future work, this aspect could be further verified by using a technique of OCT elastography, which is a technique to measure elasticity of a biological tissue from 3D OCT data [39].

### 3.4 Comparison of OCT, histology, and immunohistochemistry techniques for evaluation of the effect of exposure of hybrid catfish to glyphosate

From the above results, we can conclude that histology and immunohistochemistry data have similar characteristics. However, immunohistochemistry requires antibodies specific to antigens, making it a more complicated technique than histology. Moreover, to study the effect of exposure to glyphosate by using acetylcholinesterase (AChE) as a measure of exposure, antibodies specific to acetylcholinesterase (AChE) are required. If the antibodies are less sensitive and less specific, then the technique cannot be used.

Another important factor is the sample storage and preparation process that involves condition treatment liquid containing formalin and alcohol, which can affect the results. Sotola et al. reported on the effect of formalin and ethanol on morphology, where significant changes in body shape among fresh and formalin-fixed specimens were observed [40]. Moreover, changes in body shape continue to occur after subsequent ethanol preservation. Therefore, it is important to be aware of these morphometric changes.

In contrast with histology and immunohistochemistry, OCT requires neither chemical treatment nor fixation. It uses near-infrared light to nondestructively and noninvasively obtain cross-sectional images of biological samples as deep as 2−3 mm from the surface. The image contrast is similar to that of ultrasound images but has micrometer resolution. Furthermore, its high-speed imaging allows for 3D acquisition and 3D visualization of samples and hence allows for digital sectioning of *in vivo* tissues. *En face* images similar to what is obtained using conventional microscopy can also be digitally reconstructed. The results in this study show that OCT imaging can noninvasively reveal tissue damage in hybrid catfish that were exposed to glyphosate when compared with catfish in the control group.

Table 1 shows the comparison of several important aspects of OCT, histology, and immunohistochemistry for evaluation of the adverse effects of hybrid catfish exposed to glyphosate. Comparative characteristics include sample preparation, experimental time, instrument and equipment requirements, expertise requirements, quantity and amount of chemicals used in analysis, toxicity of chemicals used in analysis, number of samples that can be tested, and other limitations.

**Table 1:** Summary of the advantages and disadvantages among OCT, histology, and immunohistochemistry.

	OCT	Histology	Immunohistochemistry
Sample preparation	Can immediately capture 3D image of the entire bulk sample without preservation or fixation	Samples need to be preserved in preservatives such as formalin and Bouin’s fixative at least 24 h before analysis is performed.	
Experimental time	1 min for acquiring a set of 3D data and approximately 1 h for processing the results	Approximately 5-6 days for preparation and analysis. ▪ Collection and preparation of samples ▪ Immobilization in paraffin wax ▪ Sectioning to a 5-μm-thick slice ▪ Staining with Hematoxylin & Eosin (H&E) ▪ Examination under a microscope.	Approximately 5-7 days for preparation and analysis. ▪ Collection and preparation of samples ▪ Immobilization in paraffin wax ▪ Sectioning to a 5-μm-thick slice ▪ Staining with Hematoxylin & Eosin (H&E) ▪ Staining with specific antibodies ▪ Examination under a microscope.
Number of instruments and equipment used in the study	Only OCT machine	▪ Automatic tissue processor ▪ Rotary microtome ▪ Paraffin embedding center ▪ Water bath ▪ Microscope ▪ Automated vacuum tissue processor floor ▪ Digital pathology whole scanner ▪ Oven	▪ Automatic tissue processor ▪ Rotary microtome ▪ Paraffin embedding center ▪ Water bath ▪ Microscope ▪ Automated vacuum tissue processor floor ▪ Digital pathology whole scanner ▪ Oven
Expertise requirement	Specialized expertise	Specialized expertise	Specialized expertise
Chemicals used	None	▪ Bouin ▪ Fixative ▪ Paraplast ▪ Ethanol dioxane ▪ Xylene ▪ Hematoxylin ▪ Eosin ▪ Permount	▪ Bouin ▪ Fixative ▪ Paraplast ▪ Ethanol dioxane ▪ Xylene ▪ Hematoxylin ▪ Eosin ▪ Permount ▪ Gelatin ▪ Primary antibody ▪ Secondary antibody
Toxicity of chemicals used in analysis	None	Toxic chemicals such as xylene	
Number of samples that can be tested	A large number of samples can be examined at one time.		
Transverse resolution	10 μm	1 μm	1 μm
Depth resolution	10 μm	5 μm	5 μm
Imaging field of view	10 mm	0.1 mm	0.1 mm
Image contrast	Refractive index	Chemical coloring and labeling	
Other limitations of analysis	Resolution on the order of 10 μm and depth penetration on the order of 1-2 mm	Appropriate dyes and techniques must be applied to each tissue.	Antibodies used must be specific to the tissue sample and appropriate to the objectives.

## 4 Conclusion

Our study shows that OCT can be used to evaluate the effects of glyphosate contamination in freshwater fish. 3D OCT imaging is capable of capturing tissue damage caused by glyphosate even with short exposure time. According to the results, the damage was clearly observed in the brain and gill tissues. However, we found that the damage in liver tissue was barely observable. This could be due to the uniformity of structure in liver tissue that cannot be resolved by OCT imaging’s contrast.

OCT has advantages over histology and immunohistochemistry in terms of immediate assessment, less preparation time, and no chemical use. OCT can be performed immediately after the organs are extracted from fish’s body without applying any chemical process. A single dataset of 3D image acquisition of OCT can be done in less than one minute. These features allow for observation of tissue of fish’s organs as close to its original state as possible. Therefore, OCT has potential to be a powerful and novel alternative that for digital sectioning of ex vivo tissues and organs of fishes and other aquatic organisms.

## Author statement

**Chutima Thanomsit**: Conceptualization, Methodology, Resources, Investigation, Formal analysis, Writing- Original draft. **Jadsada Saetiew**: Software, Investigation, Visualization, Data curation. **Panomsak Meemon**: Conceptualization, Methodology, Funding acquisition, Resources, Supervision, Formal analysis, Writing- Reviewing and Editing.

## Declaration of Competing Interest

The authors declare that they have no known competing financial interests or personal relationships that could have appeared to influence the work reported in this paper.
